# Online dental teaching practices during the COVID-19 pandemic: a cross-sectional online survey from China

**DOI:** 10.1186/s12903-021-01547-7

**Published:** 2021-04-12

**Authors:** Zhiwei Jiang, Danji Zhu, Jialu Li, Lingfei Ren, Rui Pu, Guoli Yang

**Affiliations:** 1 The Affiliated Hospital of Stomatology, School of Stomatology, Zhejiang University School of Medicine, and Key Laboratory of Oral Biomedical Research of Zhejiang Province, Hangzhou, 310006 Zhejiang China; 2 Department of Implantology, Stomatology Hospital, School of Medicine, Zhejiang University, No.395, Yan’an Road, Xia-Cheng Region, Hangzhou, 310006 Zhejiang China

**Keywords:** Dental education, COVID-19, Online teaching

## Abstract

**Background:**

Coronavirus disease 2019 (COVID-19) emerged in China in December 2019. The COVID-19 pandemic hindered dental education, as school buildings were closed. Online dental teaching provided an alternative teaching tool for dental education. However, the efficiency of online dental teaching and student preferences for online dental teaching are unclear.

**Aim:**

To investigate the satisfaction with online dental teaching practices among undergraduate dental students and standardized resident physician training students during the COVID-19 pandemic in China.

**Methods:**

A total of 104 undergraduate dental students and 57 standardized resident physician training students from Zhejiang University participated in the study. A 12-item survey was conducted. This investigation included the teaching methods received, frequency of classes, degree of satisfaction, preferred teaching method, whether to participate in a course regarding COVID-19 prevention, and the effects of teaching. The percentages were then calculated and evaluated for each item.

**Results:**

A total of 161 students (104 undergraduate dental students and 57 standardized resident physician training students) participated in this survey. All students had online dental classes during the COVID-19 pandemic. Lecture-based learning (LBL), case-based learning (CBL), problem-based learning (PBL), team-based learning (TBL), and research-based learning (RBL) were selected as teaching methods. Students were more satisfied with LBL and CBL than PBL, RBL, and TBL. The majority of students had more than four classes per week. The most selected protective measures were hand washing, wearing masks, and wearing gloves. A total of 46.6% of students participated in courses on COVID-19. After training, the students consciously chose to wear face shields and protective clothing.

**Conclusions:**

Dental students accepted online dental learning during the COVID-19 pandemic. Students preferred LBL and CBL and were satisfied with the classes. Courses on COVID-19 helped students understand how to prevent COVID-19 transmission in the dental clinic.

## Introduction

Compared with the pathogens of the two other major respiratory diseases of the twenty-first century, severe acute respiratory syndrome (SARS) and Middle East respiratory syndrome (MERS), coronavirus disease 2019 **(**COVID-19) possesses a much higher transmission speed, which has resulted in a global pandemic reaching 100 countries and locations in a short time [1]. Moreover, the frequency of infection varied in different countries due to socioeconomic and meteorological factors [2]. Undoubtedly, convenient international travel contributed to transmission to some extent, while asymptomatic patients played a more important role in this process. The common symptoms of COVID-19 include fever, cough, fatigue, dyspnoea, myalgia, sputum production, and headache [3–6]. Moreover, some people may also experience gastrointestinal symptoms such as anorexia, diarrhoea, nausea, and vomiting [7]. However, few or even no clinical manifestations could be observed or detected in a large portion of patients [8, 9]. For the groups that inevitably contact many people daily in school, such as children and teenagers, cluster disease has been reported to be the most common form [4]. In addition, despite the low mortality rate, the high infection rate [10] and uncertain prognosis of COVID-19 remind us of the importance of prevention. Therefore, since COVID-19 was reported in December 2019 [11], dental education has faced increasing challenges. Conventional dental treatments in the Affiliated Stomatology Hospital of Zhejiang University School of Medicine were deferred, except for dental emergencies (such as acute pulpitis and tooth injuries), until June 2020. As a result, all in-person dental classes were also asked to shut down during the COVID-19 pandemic. It has been reported that many valuable solutions [12], such as online teaching, online conferencing, online lectures, and telemedicine, could be used to effectively continue medical education.

Dentists face a high risk of coronavirus infection [13]. Dental students cannot always maintain social distancing and are easily exposed to saliva and blood during dental practices. Moreover, tooth preparation and ultrasound cleaning create aerosols that have the potential to transmit viruses. Aerosols can stay airborne for a long time, increasing the risk of respiratory infection for both clinical teachers and dental students [14]. Therefore, to prevent the spread of COVID-19 among students, online dental teaching seems to be a reasonable choice. Thanks to the rapid development of technology, smart devices such as mobile phones, tablets, and laptops can provide convenient ways for students to listen to classes and contact teachers [15]. In the web-based virtual environment, students can continue to engage with live academic lectures that were previously available in classrooms [16] and can also store and review these lectures whenever and wherever they choose. On the other hand, the use of virtual patients helps dental students develop clinical skills such as patient interviewing, history taking, and symptom observation [17], which lays a foundation for their future careers.

However, teachers had no experience teaching online and did not know what the final results would be. Types of classes provided to dental students included lecture-based learning (LBL), problem-based learning (PBL), research-based learning (RBL), case-based learning (CBL), and team-based learning (TBL). LBL is a traditional and mainstream choice of education used by the majority of medical and other schools. LBL mainly consists of didactic lectures, focusing on factual knowledge delivery and memorization [18]. PBL is an educational method focused on self-directed learning, small group discussion with facilitators, and working through problems to acquire knowledge [19]. PBL is a student-centred pedagogy that can provide learners with more opportunities for applying knowledge acquired from basic science to working situations as compared to the traditional LBL method [20]. For the student, PBL emphasizes the application of knowledge and skills to the solution of problems rather than the recall of facts [21]. CBL is a long-established pedagogical method that asks students to correlate the clinical history of patients and other findings of the case to their study, aiming to encourage students to combine science learning and clinical practice [22]. TBL is a more standardized form of student-cantered teaching method. TBL carries the benefits of small-group learning, similar to CBL and PBL, but requires fewer teachers to be involved. TBL consists of three steps: advance assignment, readiness assurance tests, and team application [23]. RBL is designed to improve abilities of searching literature, to know the recent research progress in a certain field, and to find limitations in the recent studies [24]. Teachers used PBL, CBL, RBL, and TBL in online teaching practices during the COVID-19 pandemic to elevate teaching efficacy, as there is a pressing need for teaching knowledge about the prevention of COVID-19 during dental treatment. Some researchers [25] have summarized efficient measures involving hand washing and wearing masks, face shields, and gloves.

This study aimed to investigate the satisfaction with online dental teaching practices and teaching measures for preventing COVID-19 in undergraduate dental students and standardized resident physician training students and to summarize our experience.

## Materials and methods

### Participants

This study was approved by the ethics committee of the Affiliated Stomatology Hospital, Zhejiang University School of Medicine. All methods were conducted in accordance with the ethical standards of the declaration of Helsinki. All undergraduate dental students and standardized resident physician training students were invited to participate in this investigation. Out of a total of 144 senior undergraduate dental students and 205 standardized resident physician training students, 104 preclinical students and 57 standardized trainees for dental residency participated in this study according to the following inclusion criteria and exclusion criteria. Participants were asked to complete a 12-item survey. Information was collected, and the average percentages were calculated.

The inclusion criteria were as follows: (1) undergraduate dental students and standardized resident physician training students received online classes during the COVID-19 pandemic, and (2) students had electronic equipment to ensure teaching effects.

The exclusion criteria were as follows: (1) undergraduate dental students and standardized resident physician training students who did not receive online classes during the COVID-19 pandemic, (2) students who did not have internet access, and (3) junior undergraduate dental students who were not enrolled in dental classes.

### Online teaching methods

#### Online meeting applications

Two online meeting applications were the main applications used by different teaching groups at Zhejiang University School of Stomatology (ZUSS), including DingTalk (Alibaba, Hangzhou, China) and Voov Meeting (Tencent, Shenzhen, China). The apps provided free HD online meeting, live-streaming, messaging, and file-sharing systems. This allowed most of the teaching activity to be carried out online. Zhejiang University cooperated with Alibaba to develop customized versions of ZJU-Ding. All the classes and instructors were automatically organized on the app, allowing every student to remain in contact with the faculty and the school during the sudden national lockdown.

#### Online LBL

Slides and handouts were uploaded prior to the lectures. Lectures were live-streamed on the apps. Students were allowed to ask questions either by posting real-time comments or over the computer microphone, if allowed by the lecturer. Lectures were recorded at the same time to allow re-watching.

#### Online TBL

Many of the courses in ZUSS integrated TBL into their online teaching. Students were divided into groups of 3–4 individuals. Tasks such as reading, video watching, and document searching were assigned at least one week before the class. Quizzes for assuring readiness were given online at least one day before the class. Instead of focusing on factual knowledge, TBL classes spent most of the time on realistic clinical problems or in a flipped classroom format in which students were asked to give lectures on specific topics.

#### Online PBL and online CBL

The faculty of ZUSS have long-established and refined databases for PBL and CBL, both of which have been in use for offline teaching for a long time in the school. A number of teaching groups made use of these databases and conducted PBL and CBL online. To encourage collaboration, students were grouped and given handouts with different problems and cases before the class. A facilitator, typically a clinical dentist or research fellow, was assigned to each group to offer necessary guidance and help. Students were encouraged to hold and record online meetings for preparation and discussion, and participation in these meetings was taken into account when giving final grades. On the lecture day, groups were asked to give an online presentation to demonstrate their understanding of the problem or their diagnosis and treatment plan for the case. Students in other groups, facilitators, and teachers could give their opinions and questions right after the presentation by posting comments or speaking.

#### Online RBL

In RBL, students were given a research topic before classes. They were asked to search recent advances in the topic and summarize the limitation of recent studies. Moreover, students were asked to put forward their own personal opinions about future research directions. This online RBL was designed to improve abilities of searching literature, and to know the recent research progress in a certain field.

### Questionnaire

Twelve questions were designed to evaluate the effectiveness of online teaching during the COVID-19 pandemic. The basic information included student grade, gender, and age. Questions 4, 6, 9, and 11 were single-answer questions, while questions 5, 7, 8, and 12 were multiple-answer questions. The detailed information follows in Table 1. To ensure the accuracy of data collection, students were asked to submit the questionnaire twice. If the data were inconsistent, the students were asked to submit the questionnaire again.

**Table 1:** Questions of survey

Number	Question	Choice
1	Grades	Preclinical dental students/standardized trainees for dental residency
2	Gender	Male/female
3	Age	Number
4	Have online classes or not	Yes/no
5	How to take online classes	Dingding/tencent conference/others
6	Frequency of classes per week	> 4/2–4/1–2/ < 1
7	What are the forms of online classes?	LBL/PBL/CBL/RBL/TBL
8	Which kind of teaching method is more suitable for online teaching?	LBL/PBL/CBL/RBL/TBL
9	How satisfied are you with online classes?	Very satisfied/satisfied/general/ not satisfied/ very dissatisfied
10	What is your most satisfied online course? What is the teaching format?	Fill in the blank
11	Have you received training on oral consultation during the epidemic of COVID-19?	Yes/No
12	What do you think are the protective measures that dentists take in daily consultations?	Hand washing/ protection suit/face shield/goggle/mask/glove/shoe cover

COVID-19: Coronavirus disease 2019; LBL: Lecture-based learning; CBL: Case-based learning; PBL: Problem-based learning; RBL: Research-based learning; TBL: Team-based learning

### Statistical analyses

The data were collected through Questionnaire Star Software (https://www.wjx.cn). Further analyses were conducted using GraphPad Prism 8.0 (San Diego, CA) and IBM SPSS Statistics software (version 23.0). The chi-squared test was used to compare satisfaction between undergraduate students and standardized residency physician training students. The percentages of the chosen items were calculated and evaluated. The reported *P* values were two-sided, and *P* < 0.05 was considered significant.

## Results

### Participant information

A total of 161 students participated in this study, including 104 preclinical dental students and 57 standardized trainees for dental residency (107 female, and 54 male), as shown in Table 2. The average age of the participants was 22.45 years old, ranging from 19 to 30 years; 91.3% were between 20 and 25 years old.

**Table 2:** Detailed information of participants

Characteristics	Number	Proportion (%)
*Gender*		
Male	54	33.5
Female	107	66.5
*Age (years)*		
≤ 20	34	21.1
21–24	96	59.6
≥ 25	31	19.3
*Education background*		
Undergraduate students	57	35.4
Standardized residency physician training students	104	64.6

### Online teaching methods and degrees of satisfaction

**Fig. 1:**
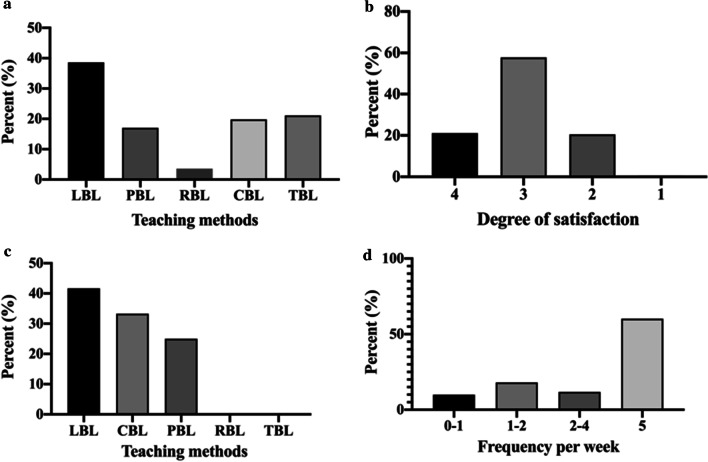
Online teaching methods and degrees of satisfaction. **a** The percentage of online teaching methods. **b** Different degrees of satisfaction of online teaching. **c** The percentage of satisfying online teaching methods. **d** The percentage of different frequency of online teaching per week

The teaching methods included LBL (38.59%), CBL (19.80%), PBL (17.00%), TBL (21.06%), and RBL (3.55%) (Fig. 1a). In Fig. 1b, 21.12% of participants were very satisfied with the courses, and 57.76% were satisfied. However, some participants thought the courses were general (20.50%) or were not satisfied (0.62%). The participants preferred LBL (41.67%) and CBL (33.33%) more than PBL (25%), TBL (0%), and RBL (0%) (Fig. 1c). In Fig. 1d, the frequency of courses per week was 60.25% (more than 4 times), 11.80% (2–4 times), 18.01% (1–2 times), and 9.94% (0–1 time). As shown in Table 3, the frequency of having online classes of undergraduate student was higher than standardized residency physician training students (92.3% vs 1.8%, *P* < 0.001).

**Table 3:** Comparison of frequency of having online class in undergraduate students and standardized residency physician training students

	Frequency of having online class (per week)		*P* value
	< 5	≥ 5	
Undergraduate students (n = 104)	8	96	< 0.001
Standardized residency physician training students (n = 57)	56	1	

The overall satisfaction rate was 78.9% (127/161). A significant difference in distribution was not observed between undergraduate students and standardized residency physician training students (Table 4). The undergraduate students were more likely to prefer LBL and TBL than standardized residency physician training students (*P* < 0.05, Table 5). The standardized residency physician training students seemed to prefer CBL more than undergraduate students did, but the difference was not significant (*P* > 0.05, Table 5).

**Table 4:** Comparison of satisfaction rates of online teaching in undergraduate student and standardized residency physician training students

Satisfying online teaching methods	Undergraduate students No. (%)	Standardized residency physician training students No. (%)	χ^2^	*P* value
TBL	39 (37.5)	7 (12.3)	11.475	< 0.001
LBL	90 (86.5)	42 (73.7)	4.120	0.042
CBL	42 (40.4)	22 (38.6)	0.049	0.825
RBL	15 (14.4)	11 (19.3)	0.646	0.503
PBL	42 (40.4)	17 (30.0)	1.769	0.232

**Table 5:** Comparison of satisfying teaching methods in undergraduate student and standardized residency physician training students

	Number of different degrees of satisfaction of online teaching				*P* value
	Not satisfied	General	Satisfied	Very satisfied	
Undergraduate students (n = 104)	0	18	63	23	0.281
Standardized residency physician training students (n = 57)	1	15	30	11	

### COVID-19 courses

**Fig. 2:**
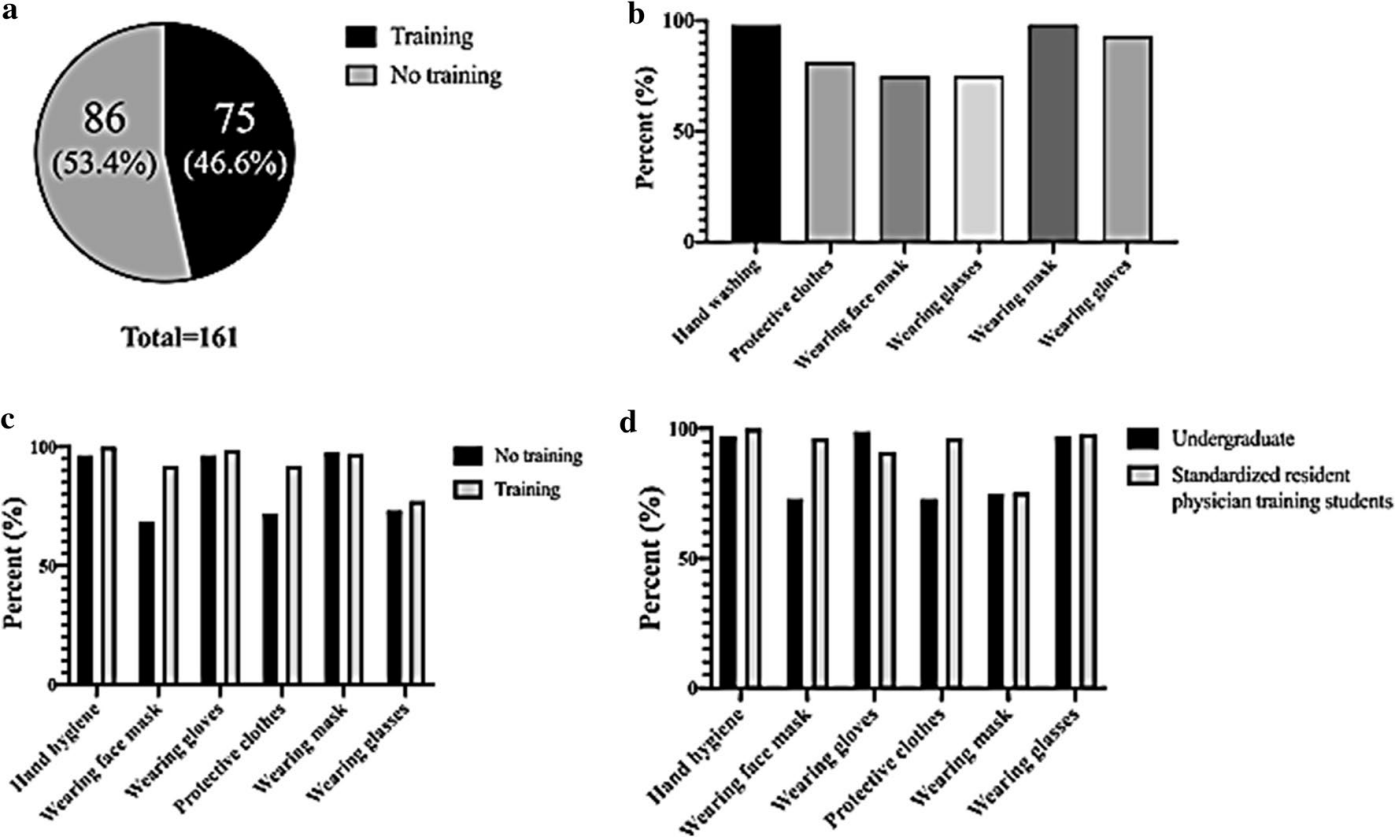
The effects of courses of COVID-19. **a** The percentage of courses and no courses in participants. **b** The percentage of protective measures chosen. **c** The percentage of different protective measures chosen after courses of COVID-19. **d** The percentage of different protective measures chosen by undergraduate students and standardized residency physician training students

According to the survey, 46.6% of participants attended COVID-19 training (Fig. 2a). More than 50% of participants thought hand washing, wearing a mask, wearing gloves, wearing protective clothes, wearing a face shield, and wearing glasses were important (Fig. 2b). After training, participants were more likely to accept wearing face shields and protective clothes (Fig. 2c). Similarly, standardized resident physician training students were more likely to choose to wear face shields and protective clothes than undergraduate students (Fig. 2d).

## Discussion

This study evaluated dental students’ satisfaction and preferences related to online dental teaching during COVID-19 at Zhejiang University. Online dental teaching provided an alternative teaching method for dental education. The COVID-19 pandemic brought us challenges and created a suitable situation for developing online teaching technology.

The United States Occupational Safety and Health Administration classified dental treatments as very high risk due to aerosol generation [26]. The greatest challenge for teachers in dental school is to decrease the risk of COVID-19 infection while ensuring the continuity and quality of dental education [27]. Online dental education was also chosen in Australia, Japan, Malaysia, Thailand, and the United States [28]. In this period, we used PBL, RBL, CBL, and TBL methods for online teaching. Students were more satisfied with the LBL and CBL methods than with the PBL and RBL methods, which was consistent with previous studies [29, 30]. One study [29] suggested online CBL as a potential teaching method that could be adopted during this period. An increasing number of studies [31] have reported that there is no significant difference between online learning and offline learning. It was found that online learning based on lectures significantly promoted the acquisition of knowledge [32]. A previous study [30] also found that online methods may be suitable to allow students to participate in CBL and that students were satisfied with the learning activity. Online teaching limited real-time eye contact and facial communication, which made teachers more uncomfortable than offline teaching [33]. Discussion in online classes is also difficult to put in practice. According to this study, students may prefer LBL. In addition, the use of dental cases may raise interest in learning and could contribute to student preference for CBL. Another study [34] proposed that group-based interprofessional education (IPE) was beneficial from a student perspective. Case-based discussions are recommended, and patient data protection should be more of a concern in this situation. Clinical applications based on dental case analysis may also be a good choice. Some educators have built a series of online courses to augment the existing educational resources [35]. During online teaching, we found that available online dental educational resources in China were limited, and more excellent online courses need to be explored.

The platforms that could be used for online teaching in China are DingTalk and Tencent Conference. Outside of China, platforms such as Skype, Google educational tools, Instagram, Facebook, YouTube, Telegram, and WhatsApp could be used. More professional apps for dental education are needed in the future. Online learning and the COVID-19 pandemic may also be considered significant stressors for students and teachers [36]. School administrators should pay more attention to the mental health of these groups. The impact of COVID-19 on dental education has been enormous. Traditional dental education should be developed to use novel and intelligent technologies for future challenges in dental education. It is suggested that dental educators from different countries or cities be invited to give presentations during the COVID-19 pandemic as a possible solution [37].

Online dental education faces particular challenges due to its dependence on hands-on training. Despite the odds, efforts have been made to overcome these challenges. An online education programme developed at the University of Nebraska built a database with digitised slides for virtual microscopy. This programme included several e-modules with an e-learning app that allowed students to observe slides and take pictures from home to practice hands-on laboratory and clinical skills [38]. These e-modules could be helpful in online courses for dental and oral pathology and oral mucosal diseases. Stephan et al. [39] integrated immersive virtual reality for teaching anatomy by reconstructing cerebral anatomy images from DICOM, CT scans, and MRIs into 3D VR formats, which achieved better engagement, more enjoyment, usefulness, and stronger learner motivation. With the development and popularization of 5G technology, applying VR in anatomy teaching could be a new trend. For prosthetic dentistry, simulation training for ceramic crown preparation was facilitated by a virtual educational system, which was found to help students improve their clinical skills [40]. Liu et al. [41] used a series of online colour training systems for dental education and found that certain exercises effectively enhanced colour sense. Computer-aided design has become widely accepted in prosthetic dentistry with precision and reproducibility similar to those of traditional wax-up methods [42]. Efforts have been made to implement CAD/CAM technology in the preclinical curriculum with satisfactory results [43]. Under the circumstance of the COVID-19 pandemic, CAD could be considered for carrying out online laboratory lessons, as it allows dentists and technicians to work on occlusion design online. Digital technologies and 3D printing are gaining increasing attention in implant dentistry and oral maxillofacial surgery for treatment planning and for guiding surgery [44]. Treatment planning and guiding plate design involve a great deal of work online and have been aided by recent advances in 3D imaging and computer-assisted planning [45]. All the work in this process could be remodelled as teaching tools. For instance, software applications for 3D printing have been used in training in dental traumatology training by Reymus et al. [46].

To further adapt clinical practise in the post COVID-19 era, courses should include how to prevent the transmission of infectious diseases. According to our investigation, some students did not know about traditional personal protective equipment and preventive measures such as hand hygiene, masks, face shields, surgical caps, gloves, and other protective clothing [28]. It has been reported that epidemiological investigation, body temperature measurements, personal protective equipment (PPE), surface disinfection, four-handed operation, and large-volume aspiration could be used to diminish the possibility of infection [47]. The majority of students in this study (98.14%) thought that wearing masks could prevent COVID-19, while percentages were lower in countries such as India (73.15%) [48], the United States (37.8%), and the United Kingdom (29.7%) [49]. However, the percentage of students who agreed with the importance of wearing face shields and protective clothing was lower among those who had not received training on the prevention of COVID-19 (68.6%) than among those in the training group (92%). Therefore, it is essential to provide courses related to COVID-19.

This study lacked feedback concerning long-term online teaching practices. This investigation did not cover technological difficulties, which may influence student satisfaction with classes, although this is not effectively related to the pedagogical methodology itself but to the transition to the online mode. Post-COVID-19, future dental education can combine online and offline classes to elevate teaching efficacy. More creative methods, such as PBL, RBL, CBL, and TBL, are needed to further increase teaching satisfaction. Courses covering the prevention of COVID-19 are suggested to respond to future infectious diseases.

## Conclusions

Dental students accepted online dental learning during the COVID-19 pandemic. Students preferred LBL and CBL and were satisfied with the classes. Courses on COVID-19 helped students understand how to prevent COVID-19 transmission in the dental clinic.

## Data Availability

The datasets generated during and analyzed during the current study are available due to they are supplied by Guoli Yang under license but are available from the corresponding author on reasonable request.

## Abbreviations

COVID-19: Coronavirus disease 2019
LBL: Lecture-based learning
CBL: Case-based learning
PBL: Problem-based learning
RBL: Research-based learning
TBL: Team-based learning
SARS: Severe acute respiratory syndrome
MERS: Middle East respiratory syndrome
IPE: Interprofessional education
PPE: Personal protective equipment
